# Use of an Industrial Tungsten Carbide Drill in the Treatment of a Complex Fracture in a Patient with Severe Osteopetrosis: A Case Report

**DOI:** 10.5704/MOJ.1703.007

**Published:** 2017-03

**Authors:** R Kunnasegaran, YH Chan

**Affiliations:** Department of Orthopaedics, Tan Tock Seng Hospital, Singapore

**Keywords:** osteopetrosis, neck of femur fracture, tungsten carbide

## Abstract

The treatment of fractures in osteopetrosis can be complicated and difficult. We describe the use of an industrial grade tungsten carbide drill bit in the treatment of one of these complex fractures. An industrial grade tungsten carbide drill bit was used to fashion a medullary canal in the surgical treatment of a left peri-implant fracture of the neck of femur in a patient with osteopetrosis. The patient was successfully treated with a hemiarthroplasty with good functional outcomes. A tungsten carbide drill bit serves as an effective and safe option in the treatment of osteopetrotic femoral neck fractures.

## Introduction

Osteopetrosis was first described in a patient with multiple fractures and osteosclerosis, in 1904 by the German radiologist Albers-Schonberg. Also known as Albers-Schonberg disease or marble bone disease, osteopetrosis is a heritable disorder characterized by defective osteoclasts. This results in a malfunction of bone resorption, leading to brittle and hard bone. Improper acidification of bone, which is required for effective osteoclastic resorption, has also been described in the pathogenesis of osteopetrosis^1^.

The compromised remodeling then predisposes the patient to fractures, fracture non-union, coxa vara, osteoarthritis, spondylosis and osteomyelitis. There is also obliteration of medullary canals and cranial nerve foramina, giving rise to a plethora of neurologic and haematologic symptoms. The quality of the bone causes many intraoperative and postoperative difficulties because of the resistance to drilling, the tendency for heat necrosis and fracture with minimal trauma. This may lead to increased blood loss and increased surgical time.

Arthroplasty is considered for osteoarthritis, fractures and periarticular non-unions, which may be recalcitrant to other treatment options. However, there is limited literature on the technical difficulties and pitfalls of this procedure and the results and complications associated with arthroplasty, in patients with osteopetrosis. This case report describes the treatment of a patient with osteopetrosis who was diagnosed with a peri-implant fracture of the left hip.

## Case Report

**Fig. 1:**
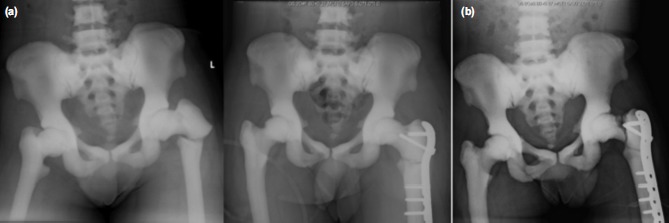
(a) Pre and post-operative radiographs following the fall in 2011 and (b) Left neck of femur fracture sustained in 2014.

The patient was an ambulant male who was first diagnosed with osteopetrosis at the age of nine following a left humeral shaft fracture. He first presented to our institution in 2011 at the age of 36 after a fall, sustaining a left subtrochanteric fracture, which was surgically treated with a proximal femoral locking plate (Fig. 1a). There were significant difficulties intraoperatively, particularly with drilling as a consequence of the quality of the bone, resulting in three broken drill bits, thermal necrosis and significant difficulty in the insertion of screws.

He presented to us again in September 2013, age 38, with sudden onset of left hip pain and inability to weight bear. Radiographs of the left hip showed a fracture at the tip of the screws within the femoral neck (Fig. 1b). Inflammatory markers were normal and a computed tomographic scan of the left hip, performed to assess for union, showed a healed fracture at the subtrochanteric region.

A bipolar hemi-arthroplasty was chosen over a total hip replacement to avoid the prolonged operative time and the increased blood loss that may be associated with the total hip replacement in such a patient, and circumvent the problems that may arise from having to ream the dense, sclerotic acetabulum and having to drill holes for the screws for cup fixation.

Our main concern was the complete obliteration of the femoral medullary canal that was seen on the radiographs of the left hip. After a thorough review of the available literature, it was decided that the best option would be to use a tungsten carbide drill bit to fashion a medullary canal for the femoral stem. An 8.4mm tungsten carbide drill bit was obtained from an engineering company specializing in cutting tools, mining and construction. With permission obtained from the Health Science Authority of Singapore and from the patient, the drill bit was sterilized and the patient was scheduled for surgery.

Intra-operatively, the proximal femoral locking plate was removed. Union of the previous fracture site was seen. As expected, there was complete obliteration of the femoral medullary canal together with thermal necrosis of the shaft and the femoral head from the previous surgery (Fig. 2a). The 8.4mm tungsten carbide drill bit was used to fashion a medullary canal with the guide of intraoperative imaging to avoid cortical perforation (Fig. 2b and 2c). Constant irrigation was used to limit the extent of thermal necrosis during the drilling process. A size 9 cemented Versys Heritage dysplastic hip stem was inserted. Cortical strut allografts were used to provide added stiffness around the implant during the initial weight bearing period. These allografts were fixed in place using a greater trochanteric reattachment plate with a cable-ready system. The total operative time was 2.5 hours with a total blood loss of 300 mls.

**Fig. 2a:**
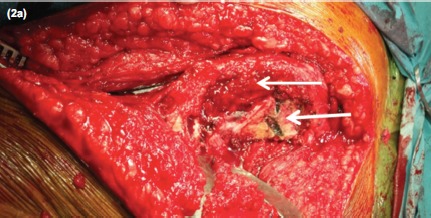
Complete obliteration of the femoral canal with evidence of thermal necrosis from the previous surgery.

**Fig. 2b:**
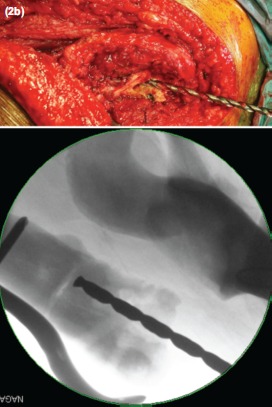
Creation of the femoral canal, using the tungsten carbide drill bit with the assistance of intra-operative imaging.

**Fig. 2c:**
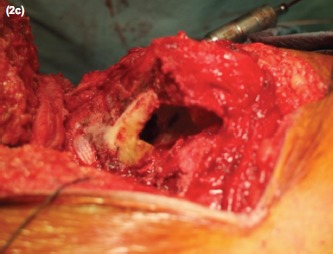
Medullary canal fashioned using the tungsten carbide drill bit.

**Fig. 3:**
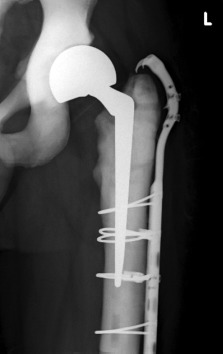
Radiograph 2 years post-operative.

The patient was started on partial weight bearing postoperatively and progressed to full weight bearing within six weeks. There were no complications two years postoperatively (Fig. 3), and the patient is currently ambulating without any significant issues or pain.

## Discussion

Surgical treatment of osteopetrotic fractures is associated with significant difficulties and pitfalls, including extreme resistance to drilling and cutting as a result of the hardness of the bone, hardware failure, peri-prosthetic fractures, refractures, delayed union, pseudoarthrosis and infection.

Histological evaluation of mature fracture callus in patients with osteopetrosis shows lack of Haversian organization with a paucity of osteoclasts up to one year after a fracture. Birmingham and Mchale^2^ reported an average time to union of 26 weeks. Peri-prosthetic and re-fracture rates are high due to altered biomechanics secondary to hardware. High complication rates, including incidence of hardware failure of 29%, rate of peri-prosthetic failure of 14%, rate of reoperation of 29% were reported in a cohort series by Birmingham and Mchale^2^.

There is limited literature on primary total hip replacement or hemi-arthroplasty for osteopetrotic femoral neck fractures. Most authors recognize the potential difficulties associated with a tight or obliterated femoral canal and usually opt for a smaller and shorter femoral stem, thus reducing the diameter and length of the medullary canal that must be created^3^. Some authors described the need for intraoperative stem shortening^4^. Jones and Hung^4^ reported difficulty fashioning a medullary canal, breaking two drill bits in the process and finally succeeding, using power reamers. Matsumo^3^ opted for a high-speed burr for the preparation of the femoral canal in three total hip replacements.

The metallic properties of tungsten carbide make it an ideal tool to create the medullary canal. It is harder, has a greater resistance to wear and is less prone to fracturing than stainless steel. Ramiah *et al*^5^ described the use of the tungsten carbide drill bit for the fashioning of the medullary canal in a patient with a peri-implant femoral neck fracture. Thermal necrosis is a recognized problem with the surgical management of these patients regardless of the modality of treatment. The tungsten carbide drill, although also associated with thermal necrosis, reduces surgical time and may potentially reduce the extent of necrosis.

The use of both cemented and uncemented stems have been reported, although cemented stems seem to be the favored option. Strickland and Berry^1^ performed total joint arthroplasties in five patients and reported stable implant fixation with cementation despite the dense sclerotic bone that may impede interdigitation of cement. Although there was little radiological follow-up information, there was only one possible case of femoral component loosening^3^.

The majority of the available literature describes the use of a total hip replacement in the treatment of this group of patients. The decision to perform a hemi-arthroplasty in our patient was made with the potential complications in mind, including the difficult reaming of the dense sclerotic acetabulum, the potentially significant blood loss and prolonged surgical time. Strickland and Berry^1^ described an average operating time of five hours for each of the three total hip replacements he performed. However, although our patient is progressing well with minimal hip pain at two years post-operatively, it is difficult to comment on the efficacy of the hemi-arthroplasty in view of the short followup period.

The few previously published reports propose possible treatment options for femoral neck fractures in patients with osteopetrosis and the challenges associated with the surgical treatment of these patients, including the fashioning of the femoral canal for the insertion of the femoral stem. Our case supports the potentially better alternative to stainless steel drill bits and reamers in the tungsten carbide drill bit as proposed by Ramiah *et al*^5^. However, further studies are needed to help reach a consensus on the optimal treatment of these patients.

## Acknowledgement

This article was put together with the written informed consent of the patient for print and electronic publication of this case report. The authors thank the patient for his permission to use his case to highlight the issues noted in this article and the use of his radiographs to demonstrate these issues.

## Conflict of Interest

The authors declare that there is no conflict of interests regarding the publication of this paper.

## References

[1] Strickland JP, Berry DJ (2005). Total joint arthroplasty in patients with osteopetrosis: a report of 5 cases and review of the literature. J Arthroplasty..

[2] Birmingham P, Mchale KA (2008). Treatment of subtrochanteric and ipsilateral femoral neck fractures in an adult with osteopetrosis. Clin Orthop Relat Res..

[3] Matsumo T (1997). Osteopetrosis and total hip arthroplasty. Int Orthop..

[4] Jones DPG, Hung NA (2004). Bilateral, uncemented total hip arthroplasty in osteopetrosis. J Bone Joint Surg Br..

[5] Ramiah RD, Baker RP, Bannister GC (2006). Conversion of failed proximal femoral internal fixation to total hip arthroplasty in osteopetrotic bone. J Arthroplasty..

